# Molecular breast cancer subtypes and therapies in a public hospital of Northeastern Brazil

**DOI:** 10.1186/1472-6874-14-110

**Published:** 2014-09-12

**Authors:** Ana Cláudia de Macêdo Andrade, Carlos Alberis Ferreira Júnior, Beatriz Dantas Guimarães, Ana Waleska Pessoa Barros, Gibran Sarmento de Almeida, Mathias Weller

**Affiliations:** 1 Centro de Ciências Biológicas e da Saúde (CCBS), Universidade Estadual da Paraíba (UEPB), Campina Grande, Brazil; 2 Programa de Pós-Graduação em Saúde Pública, Universidade Estadual da Paraíba (UEPB), Rua Juvêncio Arruda, S/N Campus Universitário (Bodocongó), CEP. 58.109 - 790 Campina Grande, Paraíba, Brazil

**Keywords:** Invasive breast cancer, Molecular subtypes, Therapeutic opportunities, Population of African, European and Indigenous ancestry, Northeastern Brazil

## Abstract

**Background:**

The frequencies of molecular breast cancer subtypes vary among different human populations. The Northeastern region of Brazil has a mixed population of African, Indigenous and European ancestry. This retrospective study investigated breast cancer subtypes and applied therapies in a public hospital of Northeastern Brazil.

**Methods:**

Data of 633 patients with invasive breast cancer from 2005 to 2011 were obtained from medical records. Status of hormone receptor (HR), HER2 and Ki67 expression index of 269 out of 633 patients were used to define subtypes of Luminal A and B, HER2 and triple negative (TN) breast cancer. Expression index of Ki67 ≥ 14% was applied to distinguish Luminal A from Luminal B subtypes.

**Results:**

Overall, 185 (68.77%) and 132 (49.07%) patients showed positive hormone receptor (HR+) and positive HER2 (HER2+) tumors. The mean age ranged from 53.33 to 58.25 years for patients with tumors of Luminal B and Luminal A subtypes, respectively (p = 0.0182). In general, 67.39% of patients with TN tumors aged over 50 and 19.57% aged between 31 and 40 years (p = 0.0046). The rate of small tumors (T1: ≤ 2.0 cm) varied from 22.73% to 52.46% for TN and Luminal A subtypes (p = 0.0088). The rate of high graded (G3) tumors was increased for HER2 and TN subtypes (35.29% and 34.28%) compared to Luminal A and Luminal B subtypes (3.92% and 12.62%), respectively (p < 0.0001). The five-year survival rate ranged from 92.86% to 75.00%, for Luminal A, HER2 and TN subtypes, respectively (HR: 0.260 to 1.015; 95% CI: 0.043 to 3.594; p = 0.2589). Patients with HER2 positive (HER2+) breast tumors did not receive immunotherapy and chemotherapy application varied from 54.84% to 86.49% for Luminal A and HER2 subtypes, respectively (p = 0.0131).

**Conclusions:**

The results of this study revealed a high percentage of HER2+ breast tumors and an increased rate of patients with TN tumors aged over 50 years. This emphasizes the need for establishing immunotherapy as an additional therapeutic option to improve clinical outcomes for patients with HER2+ tumors and to investigate the risk factors of TN breast cancer.

## Background

Breast cancer represents a molecular and cellular heterogeneous group of diseases with different clinical outcomes [1]. The gene expression profile revealed that the levels of Estrogen (ER), Progesterone (PR) hormone receptors (HR) and HER2 overexpression characterize tumors of different subtypes, including Luminal A (ER + and/or PR + and HER2-), Luminal B (ER + and/or PR + and HER2+), HER2 overexpressed (ER- PR- HER2+) and triple negative (TN; ER- PR- HER2-) breast cancer [2-5]. Subtypes have different prognostic values, and Luminal A and triple negative tumors show the best and worst outcomes, respectively [6-9].

It is generally recognized that the risk factors affect the ER, PR and HER2 expression: Post-menopause state, high body mass index and increased height, were associated with positive PR tumors and breastfeeding period equal to or longer than seven months was negatively associated with TN breast cancer [10,11]. The increased frequency of positive ER breast tumors in African American, Hispanic and white women, was associated with changing incidence of obesity and parity [12]. There is considerable controversy regarding the role of ancestry in the etiology of breast cancer [13]. However, results of previous studies have indicated that variations of subtypes among populations of African, Asian and Caucasian ancestry have also a biological basis [14-16]. Americans of African ancestry have decreased incidence of breast cancer, but more frequently, they have aggressive, invasive high- grade TN tumors at younger age with increased mortality rate when compared to patients of Caucasian ancestry [13,15,17,18].

In Brazil, the ministry of health launched a public breast cancer- screening program in the year 2003 and recommended the participation of women aged 50–69 years [19]. The public “Unified Health System” (Sistema Único de Saúde; SUS) is financing the screening program and also radiotherapy and chemotherapy for breast cancer patients. The Brazilian National Institute of Cancer (INCA) expected 57,120 new cases for the year 2014 with an estimated risk of 56.09 cases per 100 thousand women [19]. In the Northeastern region of Brazil, mortality rates increased on average 5.3% every year in the last decade, while it declined slightly in other regions of the country [20]. The Brazilian population is aging and this demographic process is the combined result of increased life expectation, mainly in the Northern and Northeastern regions and decreased birth rates in the entire country [21]. Demographic development, changing lifestyle and associated risk factors may explain this drastic increase in the mortality rate observed in the Northeastern region. Biological factors could also contribute to differences observed. Populations from Southern Brazil have mainly Caucasian ancestry; while a mixed origin of African, European and Indigenous ancestry is characteristic of populations in the Northeastern region [22]. The availability of literature about breast cancer in Brazil is low [23]. To the present moment, few studies have focused on risk factors and breast cancer screening, whereas little is known about molecular breast cancer subtypes of different Brazilian populations. Patients of African ancestry had an increased frequency of TN breast cancer [24]. Another study including patients with undefined ancestry also reported increased incidence of the TN subtype [25].

The aim of the present study was to investigate the frequency of breast cancer subtypes in a population of Northeastern Brazil of mixed African, European and Indigenous ancestry. The objective was to analyze associated clinical and histopathological characteristics of subtypes and to investigate the currently applied therapies.

## Methods

### Data sampling

The data sampling protocol was reviewed and approved by the Brazilian National Research Ethics Committee (CONEP; Nr.: CEP-UEPB: 0239.0.133.000-12).

Registered data from pathological reports of medical records were obtained from the “Fundação de Assistência da Paraíba” (FAP) public hospital of Campina Grande, Paraíba, Brazil. Patients of the FAP hospital are representative for the state of Paraíba, with a population of mixed African, European and native Indigenous ancestry. Information about ancestry was obtained by the self- identification method. Patients were asked if they have European or any kind of mixed African, Indigenous and European ancestry.

Registered data were obtained from 633 female patients with confirmed diagnosis of invasive breast cancer. Data were sampled between March and November 2013. Data of *in situ* breast carcinoma were not sampled. Data from patients with incomplete immunohistochemistry analysis of hormone receptor and/or HER2/neu expression were excluded from the study. Only data from patients with complete immunohistochemistry analysis were sampled in the medical record. Furthermore, data obtained from secondary tumors or lymph node surrogates were also excluded from the sample. Data were exclusively obtained from primary tumors before application of any chemo or radiotherapy. Data of death cases were sampled from 2005 to 2008. Complete information about hormone receptor (ER and PR), HER2/neu status and Ki67expression was obtained from 295 out of 633 patients. Data sampling included histological type of the primary tumor, its grade, size, hormonal receptor status (ER, PR), HER2/neu status, state of lymph nodes and presence or absence of distant metastases. Furthermore, data regarding age, menopause state and patients’ ancestry were collected from pathological reports of medical records.

### Tumor size and grading

Tumors were classified according to the WHO classification of breast tumors and graded with the Elston and Ellis (EE) histoprognostic grade [26]. Tumor size was categorized according to the American Joint Committee on Cancer (AJCC) as follows: T1: ≤ 2.0 cm; T2: > 2.0 cm ≤ 5.0 cm; T3: > 5.0 cm. Trained pathologists from two private laboratories performed the grading and immunohistochemistry assays of tumor specimens.

### Immunohistochemistry

Her2/neu was scored from 0 to 3; 0 = no staining; score 1 = faint, partial membrane staining; score 2 = weak complete membrane staining in > 10% of cancer cells; score 3 = intense and complete membrane staining in > 10% of cancer cells. Cases of score 2 were analyzed with HER2/neu FISH. Her2 positive cases were defined as with immunohistochemistry scores greater than or equal to 2 and FISH positive. ER and PR positivity was defined as any positive nuclear staining in ≥ 1% of tumor cells. Combinations of hormone receptor (ER, PR), HER2/neu status and Ki67 expression to define molecular breast cancer subtypes were used as follows: Luminal A: ER positive (ER+) and/or PR positive (PR+), Her2 negative (Her2-) with Ki67 ≤ 14%; Luminal B: ER positive (ER+) and/or PR positive (PR+) and Her2 positive (Her2+), and any Ki67; Luminal B: ER positive (ER+) and/or PR positive (PR+) and Her2 negative (Her2-), with Ki67 > 14%; Her2 subtype: Her2 positive (HER2+), ER negative (ER−) and PR negative (PR-) with any kind of Ki67 expression; Triple negative (TN): ER negative (ER-), PR negative (PR-) and HER2 negative (HER2-) with any kind of Ki67 expression. There was no distinction between basal-like and normal-like TN tumor subtypes. Of the 295 data sets, 26 had unspecified molecular subtype with unknown ER, PR or HER2 expression status. These unspecified 26 samples were excluded. The remaining 269 sampled data sets were from years 2005 (N = 30), 2006 (N = 40), 2007 (N = 37), 2008 (N = 34), 2009 (N = 36), 2010 (N = 45) and 2011 (N = 47).

### Statistical analyses

All statistical analyses were performed on GraphPad Prism software version 6 (La Jolla, CA). Chi-Square (*χ*^2^) and Fisher‘s exact test were applied to compare categorical variables. ANOVA and the Kruskal- Wallis test were applied to compare patients’ age. *T*-test was applied to compare pair-by-pair mean age. Cumulative survival probabilities were calculated through the Kaplan**–** Meier method. Survival rates were compared by log-rank (Mantel-Cox) test. Overall, five-year survival rate was calculated as the period from the year of diagnosis to the year of death from any cause.

## Results

Registered data of breast cancer patients revealed 269 invasive primary tumors with known ER, PR and HER2 status that belonged to one of the four defined subtypes. Of these tumors, 179 (66.54%), 152 (56.51%) and 132 (49.07%) were positive for ER, PR and HER2, respectively. Overall, 185 (68.77%) and 84 (31.23%) patients had positive (HR+) and negative hormone receptor (HR-) tumors, respectively. When positive HR status was stratified according to age, there was no significant difference between patients aged **≤**50 years (64.60%) and those aged **>** 50 years (74.07%) (p = 0.1099).

The majority of 120 (44.61%) tumors were Luminal B, 64 (23.79%) were Luminal A, 39 (14.50%) were HER2 and 46 (17.10%) were TN (Table 1). Patients had mean age of 55.36 ± 0.82 years and age ranged from 26 to 92 years (Table 1). The percentage of patient’s aged 31 to 40 and 41 to 50 years varied from 7.69% (HER2) to 19.57% (TN) and from 10.87% (TN) to 38.33% (Luminal B), respectively (p = 0.0046; Table 1). Patients with tumors of Luminal A subtype had an increased mean age of 58.25 ± 1.827 years compared to 53.33 ± 1.153 years of those with tumors of Luminal B molecular subtype (p = 0.0182; Table 1). Pre-menopause state tended to be increased for patients with tumors of Luminal B (38.89%) and TN (33.33) molecular subtypes compared to those with tumors of Luminal A (22.73%) and HER2 (26.92%) subtypes (Table 1). However, none of these differences were significant. Information about ancestry was obtained from 197 out of 269 patients. Self-identification did not lead to significant differences between molecular subtypes: Of 197 patients, 168 (85.28%) had Caucasian and 29 (14.72%) had mixed ancestry (p = 0.7646; Table 1).

**Table 1:** Molecular breast cancer subtypes by frequency and characteristics of patients (Total number N = 269)

	**Luminal A**		**Luminal B**		**HER2/neu**		**TN**		**p**
	**N**	**%**	**N**	**%**	**N**	**%**	**N**	**%**	
	**64**	**23.79**	**120**	**44.61**	**39**	**14.50**	**46**	**17.10**	
**Age categories**									
≤ 30	4	6.25	1	0.83	1	2.56	1	2.17	0.0046
31-40	5	7.81	14	11.67	3	7.69	9	19.57	
41-50	10	15.63	46	38.33	8	20.51	5	10.87	
51-60	16	25.00	27	22.5	11	28.21	12	26.09	
> 60	29	45.31	32	26.67	16	41.03	19	41.30	
**Mean age in years**^**1**^									
	58.25 ± 1.827		53.33 ± 1.153		56.59 ± 2.054		55.63 ± 2.025		0.1075
**Menopause state**									
Pre	10	22.73	28	38.89	7	26.92	10	33.33	0.3027
Post	34	77.27	44	61.11	19	73.08	20	66.67	
Missing	20		48		13		16		
**Ancestry**									
Mixed	8	16.00	14	15.73	4	16.67	3	8.82	0.7646
Caucasian	42	84.00	75	84.27	20	83.33	31	91.18	
Missing	14		31		15		12		

^1^Pair wise comparison of Luminal A and Luminal B: p = 0.0182.

**Figure 1:**
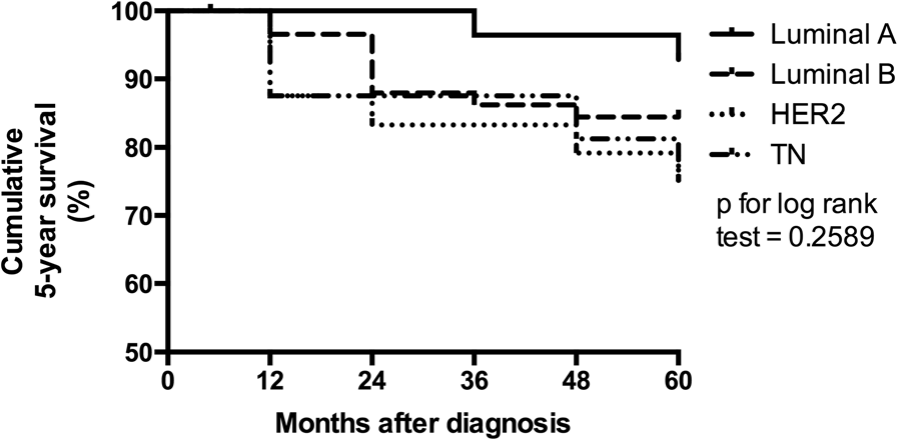
Cumulative five-year survival by molecular subtype in 127 breast cancer patients of years from 2005 to 2008.

The highest proportion (52.63%) of tumors ≤ 2 cm (T1) detected was Luminal A, and the lowest (22.73%) of TN subtype (p = 0.0088). Intermediate size tumors > 2.0 cm ≤ 5.0 cm (T2) were most frequent (70.45%) in the TN subtype (Table 2). The size category of Luminal B tumors was slightly and significantly different from TN tumors (p = 0.0448; Table 2). Lymph node status and presence or absence of metastases was not significantly different among subtypes (Table 2). However, the highest percentage (51.35%) of positive lymph node status was found in the group of patients with TN tumors (Table 2). High graded (G3) tumors were more frequent (35.29% and 34.28%) in HER2 and TN subtypes compared to Luminal A and Luminal B subtypes (3.92% and 12.62%), respectively, and in the case of low graded (G1) tumors, the opposite was observed (p < 0.0001; Table 2). Triple negative tumors showed the highest mean percentage of Ki67 expressing cells (p < 0.0001; Table 2).Kaplan- Meier analysis of the five-year survival rate of Luminal A, Luminal B, HER2 and TN molecular subtypes were 92.86%, 84.75%, and the latter 75.00% each, respectively (HR: 0.260 to 1.015; 95% CI: 0.043 to 3.594; p = 0.2589; Figure 1).

**Table 2:** Molecular breast cancer subtypes by tumor histopathology (Total number N = 269)

	**Luminal A**		**Luminal B**		**HER2/neu**		**TN**		**p**
	**N**	**%**	**N**	**%**	**N**	**%**	**N**	**%**	
**Tumor size, cm** (T category; T1: ≤ 2.0 cm; T2: > 2.0 cm ≤ 5.0 cm; T3: > 5.0 cm)^**1**^									
T1	32	52.46	49	41.88	14	35.90	10	22.73	0.0803
T2	26	42.62	57	48.72	22	56.41	31	70.45	
T3	3	4,92	11	9.40	3	7.69	3	6.82	
Missing	3		3		0		2		
**Lymph node status**									
Positive	22	43.14	41	40.20	13	41.94	19	51.35	0.7067
Negative	29	56.86	61	59.80	18	58.06	18	48.65	
Missing	13		18		8		9		
**Distant metastases**									
Positive	9	16.36	14	12.73	6	18.18	8	17.78	0.7922
Negative	46	83.64	96	87.27	27	81.82	37	82.22	
Missing	9		10		6		1		
**Histological grade**									
Low (G1)	11	21.57	14	13.59	1	2.94	1	2.86	< 0.0001
Inter. (G2)	38	74.51	76	73.79	21	61.77	22	62.86	
High (G3)	2	3.92	13	12.62	12	35.29	12	34.28	
Missing	14		17		5		11		
**Ki67 positive cells (%)**									
Mean	6.05 ± 0.467		36.71 ± 1.819		36.43 ± 4.120		50.38 ± 4.691		< 0.0001

^1^Pair wise comparison of Luminal A and TN: p = 0.0088; Luminal B and TN: p = 0.0448.

Complete data for histological staging were available for 212 out of 269 breast tumors. The histological stage of tumors was not significantly different among subtypes (p = 0.4960; Table 3). TN subtype had a decreased rate of stage IA tumors (10.26%) and a comparable rate of stage IV tumors (20.51%) as Luminal A subtype (Table 3). Of the 269 patients, 260 received chemo, radio and/or hormone therapy in the hospital (Table 3). The chemotherapeutic regime included cyclophosphamide, doxorubicin and fluorouracil. Hormone therapy was based on tamoxifen and anastrozole. Patients with HER2+ tumors did not receive Trastuzumab or Pertuzumab- based immunotherapy. Patients with HER2 subtype tumors received preferentially chemotherapy (86.49%; p = 0.0131; Table 3). Among patients with TN breast cancer, 16 (37.21%) did not receive any chemotherapy (Table 3). The percentage of patients who received radiotherapy varied from 79.03% to 97.67% for Luminal A and TN subtypes, respectively (p = 0.0462; Table 3). Hormone therapy was applied in 75.81% and 82.20% of patients with tumors of Luminal A and B subtypes, respectively (p < 0.0001; Table 3).

**Table 3:** Molecular breast cancer subtypes by histological stage and applied therapies (Total number N = 212)

	**Luminal A**		**Luminal B**		**HER2**		**TNBC**		**p**
	**(N = 64)**		**(N = 120)**		**(N = 39)**		**(N = 46)**		
	**Nr**	**%**	**Nr**	**%**	**Nr**	**%**	**Nr**	**%**	
	**Stage**								
IA	12	26.67	29	29.29	6	20.69	4	10.26	0.4960
IIA	13	28.89	34	34.35	7	24.14	14	35.90	
IIB	8	17.78	16	16.16	9	31.03	10	25.64	
IIIA	2	4.44	6	6.06	1	3.45	3	7.69	
IIIC	1	2.22	-	-	1	3.45	-	-	
IV	9	20.00	14	14.14	5	17.24	8	20.51	
Total	45		99		29		39		
n/a	19		21		10		7		
	**Adjuvant chemotherapy**								
Yes	34	54.84	72	61.02	32	86.49	27	62.79	0.0131
No	28	45.16	46	38.98	5	13.51	16	37.21	
	**Radiotherapy**								
Yes	49	79.03	99	83.90	33	89.19	42	97.67	0.0462
No	13	20.97	19	16.10	4	10.81	1	2.33	
	**Hormone therapy**								
Yes	47	75.81	87	82.20	-	-	-	-	<0.0001
No	15	24.19	21	17.80	37	100.0	43	100.0	
n/a	2		2		2		3		

n/a = not available.

## Discussion

Missing immunohistochemistry assays can be the result of advanced phase of the disease, excluding the advantage of any chirurgic intervention. Additionally, patients of the present study had to cover the costs of immunohistochemistry assays on their own as the public FAP hospital does not offer it and the public health system does not cover the cost of private laboratories. This may lead low-income patients not to afford for immunohistochemistry assays. Originally, 283 data sets with and without HR and HER2 expression status were obtained from years 2005 to 2008 including 63 (22.26%) death cases within five years. Immunohistochemistry assays were only available for 22 (34.92%) out of the 63 death cases. In view of the great amount of missing data, the bias effect on the survival analysis is very probable and incomplete information represents a general limitation of this retrospective study. Furthermore, the intergroup heterogeneity among patients with tumors of one of the four molecular subtypes may have accounted for statistical bias of clinical data analysis.

Previous studies have shown that patients with positive HR (ER + and/or PR+) tumors of Luminal A subtype and a Ki67 index > 14% have poorer outcomes, compared to those with Ki67 index ≤ 14% [27]. Furthermore, the outcome of Luminal A subtype with Ki67 index > 14%, was comparable to HER2+ tumors of Luminal B subtype. For this reason, several studies classified Luminal A tumors with high expression of Ki67 as Luminal B subtype [27-29]. The present study identified Luminal B subtype as the most frequent subtype (44.61%). This result is similar to studies with Chinese and North-African populations that applied Ki67 index > 14% as criteria to distinguish Luminal A from Luminal B subtypes [28,29]. The study of El Fatemi (2012) identified Luminal B subtype for 41.01% of all cases studied [29]. The percentage of TN breast tumors observed (17.10%) was consistent with previous studies that revealed frequencies ranging from 9.00% to 30.60% for different populations [13,18,30-36].

Results revealed an extraordinary high percentage of HER + tumors (49.07%), if compared to populations from Spain (19.50%), China (22.10% and 39.60%), Mali (18.00%), and Hispanic women living in the United States younger than 50 years of age (23.50%) [11,15,28,37,38]. Corresponding to this high percentage of HER2+ tumors, results revealed an increased frequency (14.50%) of HER2 subtype, compared to data obtained for Western Europe (5.2%), Western Africa (9.00%), Northern Africa (9.20%), China (9.0% and 13.70%), and Afro-American populations (10.10%) [11,15,28,29,37,38].

Consistent with other studies, patients aged ≤ 40 had an increased rate (19.57%) of TN tumors [13,18,34,39]. A high percentage of TN tumors belonged to age groups from 51 to 60 (26.09%) and > 60 (41.30%) years. Previous studies with Afro American and Hispanic women living in the US revealed that 29.50% and 19.40% of TN tumors, respectively, were detected in patients aged over 50 years [15]. Other studies with populations from Northern and Sub Saharan Africa revealed frequencies of 35.00% and 44.90% for patients aged over 50 years with tumors of basal-like subtype, respectively [39,40]. This means that the results of TN tumors indicated an extraordinary high percentage (67.39%) of patients aged over 50 years. In general, patients had mean age of 55.36 years, while previous studies reported a mean age of 51.5 and younger [15,39,40]. This could lead to a bias in the present data, increasing the rate of TN tumors towards older age groups.

Different studies have shown that patients with tumors of TN subtype had the lowest mean age [6,34]. In contrast, the present study revealed the lowest mean age of 53.33 years for Luminal B subtype. This was most obvious for the age group from 41 to 50 years, which included 38.33% of all patients with tumors of Luminal B subtype. Other studies that have applied the Ki67 index to define the luminal subtype (A or B) have shown that the lowest mean age was not found for patients with TN tumors, but in those with luminal B tumors, and the highest mean age was found for patients with luminal A tumors [28,40]. Consistent with present results, the study of Xue and colleagues (2012) revealed the highest and lowest rate of pre-menopause state for Luminal B instead of TN and Luminal A subtypes, respectively [28].

There was no significant difference among subtypes as a function of ancestry, and many previous studies revealed associations between breast cancer subtypes and geographic origin of populations [13,15,17,39]. It is important to point out that self-identification does not necessarily reflect real ancestry. On the one hand, as the population is highly mixed, persons who identify themselves as Caucasian may also have African and/or Indigenous ancestry and vice versa. Therefore, the mixture of populations with different geographic origins may obscure biological differences between them. On the other hand, the present result has to be interpreted with care, as there was a very low number of patients with mixed ancestry and known HR, respectively, HER2 status (N = 29).

Recent studies have revealed that the increased tumor size of TN breast tumors was associated with decreased overall survival and increased recurrence rate [41,42]. Present data have indicated a significant increase in tumor size of TN breast cancer compared to Luminal A and B molecular subtypes. This increase was mainly due to a high rate of intermediate size tumors > 2.0 cm ≤ 5.0 cm (T2: 70.45%) and a decrease rate of small size tumors ≤ 2.0 cm (T1: 22.73%) compared to Luminal A and B subtypes. This result is consistent with previous findings that attributed increased tumor size of TN subtype also to low and high rates of T1 and T2 categories, respectively [15,36,40].

Present data about lymph node status and distant metastases did not indicate a clear difference between TN tumors and other subtypes. Similarly, previous studies did not identify a clear association between TN subtype and lymph node status or distant metastasis [27,37,42,43]. Several studies revealed the lowest (34.00%- 52.40%) and highest percentage (56.00%- 86.70%) of positive lymph node status for Luminal A and HER2 molecular subtypes, respectively [13,28,29]. In contrast, another study with Afro-American and Hispanic women revealed the highest percentage (60.20%) of positive lymph node status for TN molecular subtype [15]. Present results also revealed the highest percentage (51.35%) of positive lymph node status for TN molecular subtype. Differences among the other three molecular subtypes were small. Therefore, increased positive lymph node status of the TN subtype could indicate a real biological difference, whereas a sampling artifact, due to the low number of data, may obscure further differences among molecular subtypes.

Tumors of HER2 and TN subtypes were higher graded (G3: 35.29% and 34.28%) than those of Luminal A and B subtypes (G3: 3.92% and 12.62%). Additionally, TN tumors had on average a high index of Ki67 positive cells (50.38%), which is associated with increased aggressiveness of TN breast tumors [44]. A high grade and proliferation rate of TN tumors has been reported in many previous studies [15,28,37,42,43]. In the case of HER2 subtype, this relationship is not so clear: Some studies have revealed a higher grade of HER2 subtype compared to Luminal A and B tumors [28,45], but other studies did not identify this relationship [15,40].

The Brazilian public health system does not cover immunotherapy costs for HER2 positive breast cancer. This may explain the high percentage of patients with HER2 positive breast tumors who received chemotherapy (86.49%). Brazilian health centers, with few exceptions in cities like São Paulo and Rio de Janeiro, also do not offer tests for breast cancer patients to analyze the potential benefit of chemotherapy. In the present study for example, at least 128 (47.58%) out of 269 patients had invasive stage I or II tumors smaller than five centimeters in size with less than four metastasized lymph nodes. They would have been eligible for the MammaPrint test [46,47].

Advanced stages of the disease or age could advocate against chemotherapeutic intervention. Among the 16 (37.21%) TN breast cancer patients who did not receive chemotherapy, only three had stage IV, 13 had stage IA, IIA or IIB breast cancers and only five of these aged over 50 years. As chemotherapy is one of the few therapeutic options for TN breast cancer, it remained unclear why all these patients received radiotherapy and no chemotherapy. Similarly, it was not clear why 15 (24.19%) and 21 (17.80%) patients with positive HR breast tumors of Luminal A and B molecular subtypes did not receive hormone therapy.

## Conclusions

The application of the Ki67 index > 14% as criteria to distinguish Luminal A from Luminal B molecular subtypes lead to an increased frequency of Luminal B breast cancer. Furthermore, patients with tumors of Luminal B molecular subtype had the lowest mean age. These results of breast cancer patients in Northeastern Brazil were consistent with previous studies with populations from China and Northern Africa that also applied the Ki67 index > 14% as criteria. Comparable to previous findings, tumors of TN molecular subtype were also higher graded, had high Ki67 expression index and increased size compared to breast tumors of Luminal A and B molecular subtype.

In contrast to studies with other populations, the present results revealed a high rate of patients with TN tumors aged over 50 years. To date, there are no studies about the risk factors that increase the frequency of TN breast cancer in populations from Northeastern Brazil. Future studies should aim on the identification of such potential risk factors. This could help to improve ongoing prevention programs to reduce the incidence of this aggressive form of breast cancer. Furthermore, data indicated an increased frequency of patients with positive HER2 and high graded tumors of HER2 molecular subtype, respectively. These patients did not receive immunotherapy. This emphasizes the need to improve positive HER2 breast cancer treatment in Northeastern Brazil through the application of immunotherapy as additional therapeutic option. Therapeutic opportunities could also be further improved through the application of tests with molecular markers to predict the benefits of chemotherapy.

## Abbreviations

ER: Estrogen receptor; HR: Hormone receptor; PR: Progesterone receptor; TN: Triple negative.

## Competing interests

The authors declare that they have no interests that compete with any of the contents of the manuscript.

## Authors’ contributions

AA, CJ, BG, AB and GS equally contributed to conception, data acquisition and critical review of the manuscript. MW participated in the study design and manuscript draft. All authors read and approved the final manuscript.

## Pre-publication history

The pre-publication history for this paper can be accessed here:

http://www.biomedcentral.com/1472-6874/14/110/prepub

## References

[B1] Rakha EA Pitfalls in outcome prediction of breast cancer J Clin Pathol 2013 66 458 464 10.1136/jclinpath-2012-201083 23618694

[B2] Cancer Genome Atlas Network Comprehensive molecular portraits of human breast tumors Nature 2012 490 61 70 10.1038/nature11412 23000897 PMC3465532

[B3] Sørlie T Tibshirani R Parker J Hastie T Marron JS Nobel A Deng S Johnsen H Pesich R Geisler S Demeter J Perou CM Lønning PE Brown PO Børresen-Dale AL Botstein D Repeated observation of breast tumor subtypes in independent gene expression data sets PNAS 2003 100 8418 8423 10.1073/pnas.0932692100 12829800 PMC166244

[B4] Wirapati P Sotiriou C Kunkel S Farmer P Pradervand S Haibe-Kains B Desmedt C Ignatiadis M Sengstag T Schütz F Goldstein DR Piccart M Delorenzi M Meta-analysis of gene expression profiles in breast cancer: toward a unified understanding of breast cancer subtyping and prognosis signatures Breast Cancer Res 2008 10 R65 10.1186/bcr2124 18662380 PMC2575538

[B5] Perou CM Sørlie T Eisen MB Van de Rijn M Jeffreyk SS Rees CA Pollack JR Ross DT Johnsen H Akslen LA Fluge Ø Pergamenschikov A Williams C Zhu SX Lønning PE Børresen-Dale AL Brown PO Botstein D Molecular portraits of human breast tumors Nature 2000 406 747 752 10.1038/35021093 10963602

[B6] Cadoo KA Fornier MN Morris PG Biological subtypes of breast cancer: current concepts and implications for recurrence patterns Q J Nucl Med Mol Imaging 2013 57 312 321 24322788

[B7] Blows FM Driver KE Schmidt MK Broeks A Van Leeuwen FE Wesseling J Cheang MC Subtyping of breast cancer by immunohistochemistry to investigate a relationship between subtype and short and long term survival: a collaborative analysis of data for 10,159 cases from 12 studies Plos Med 2010 7 e1000279 10.1371/journal.pmed.1000279 20520800 PMC2876119

[B8] Rakha EA Reis-Filho JS Ellis IO Combinatorial biomarker expression in breast cancer Breast Cancer Res Treat 2010 120 293 308 10.1007/s10549-010-0746-x 20107892

[B9] Onitilo AA Engel JM Greenlee RT Mukesh BN Breast cancer subtypes based on ER/PR and Her2 expression: comparison of clinicopathologic features and survival Clin Med Res 2009 7 1–2 4 13 19574486 10.3121/cmr.2009.825 PMC2705275

[B10] Rosner B Glynn RJ Tamimi RM Chen WY Colditz GA Willett WC Hankinson SE Breast cancer risk prediction with heterogeneous risk profiles according to breast cancer tumor markers Am J Epidemiol 2013 178 2 296 308 10.1093/aje/kws457 23645624 PMC3816337

[B11] Redondo CM Gago- Domínguez M Ponte SM Enguix Castelo M Jiang X Alonso García A Peña Fernández M Ausencia Tomé M Fraga M Gude F Martínez ME Muñoz Garzón V Carracedo A Castelao JE Breast feeding, parity and breast cancer subtypes in a Spanish cohort PLoS One 2012 7 e40543 10.1371/journal.pone.0040543 22792365 PMC3394701

[B12] DeSantis C Ma J Bryan L Jemal A Breast cancer statistics 2013 CA Cancer J Clin 2014 64 1 52 62 10.3322/caac.21203 24114568

[B13] Carey LA Perou CM Livasy CA Dressler LG Cowan D Conway K Karaca G Troester MA Tse CK Edmiston S Deming SL Geradts J Cheang MCU Nielsen TO Moorman PG Shelton Earp H Millikan RC Race, breast cancer subtypes, and survival in the Carolina breast cancer study JAMA 2006 295 2492 2502 10.1001/jama.295.21.2492 16757721

[B14] Ambrosone CB Young AC Sucheston LE Wang D Yan L Liu S Tang L Hu Q Freudenheim JL Shields PG Morrison CD Demissie K Higgins MJ Genome-wide methylation patterns provide insight into differences in breast tumor biology between American women of African and European ancestry Oncotarget 2014 5 237 248 24368439 10.18632/oncotarget.1599 PMC3960204

[B15] Wu Y Sarkissyan M Elshimali Y Vadgama JV Triple negative breast tumors in African-American and Hispanic/Latina women are high in CD44+, low in CD24+, and have loss of PTEN PLoS One 2013 8 e78259 10.1371/journal.pone.0078259 24167614 PMC3805609

[B16] Chen M Xu R Turner JW Warhol M August P Lee P Race and the molecular origins of breast cancer in Chinese women: breast cancer in Chinese women Ann Surg Oncol 2012 19 4085 4093 10.1245/s10434-012-2452-x 22732838

[B17] Clarke CA Keegan THM Yang J Press DJ Kurian AW Patel AH Lacey Jr JV Age-specific incidence of breast cancer subtypes: understanding the black–white crossover JNCI 2012 104 1094 1101 10.1093/jnci/djs264 22773826 PMC3640371

[B18] O’Brien KM Cole SR Tse CK Perou CM Carey LA Foulkes WD Dressler LG Geradts J Millikan RC Intrinsic breast tumor subtypes, race, and long-term survival in the Carolina breast cancer study Clin Cancer Res 2010 24 6100 6110 21169259 10.1158/1078-0432.CCR-10-1533 PMC3029098

[B19] Instituto Nacional de Câncer (INCA) Estimativa 2014: Incidência de câncer no Brasil[http://www.inca.gov.br/estimativa/2014/]

[B20] Freitas-Junior R Reis Gonzaga CM Aires Freitas NM Martins E de Cássia de Maio Dardes R Disparities in female breast cancer mortality rates in Brazil between 1980 and 2009 Clinics 2012 67 731 737 10.6061/clinics/2012(07)05 22892915 PMC3400161

[B21] Instituto Brasileiro de Geografia e Estatística (IBGE) Censo 2010 [http://www.censo2010.ibge.gov.br/]

[B22] Salzano FM Bortolini MC The Evolution and Genetics of Latin American Populations 2002 New York: Cambridge University Press

[B23] Lee BL Liedke PE Barrios CH Simon SD Finkelstein DM Goss PE Breast cancer in Brazil: present status and future goals Lancet Oncol 2012 13 e95 e102 10.1016/S1470-2045(11)70323-0 22381937

[B24] Brito Corrêa P Pereira Toralles MB Abe-Sandes K Bonfim Machado TM Ferreira Bonfim T Meyer L Abe-Sandes C Nascimento R Câncer de mama triplo negativo e sua associação com ancestralidade africana Rev Ci Med Biol 2010 9 Supl.1 3 7

[B25] Viegas de Carvalho L Pereira EM Frappart L Boniol M Marques Bernardo W Tarricone V Tavtigian S Southey MC Molecular characterization of breast cancer in young women Rev Assoc Med Bras 2010 56 3 278 287 20676533 10.1590/s0104-42302010000300010

[B26] Elston CW Ellis IO Pathological prognostic factors in breast cancer: the value of histological grade in breast cancer: experience from a large study with long-term follow-up Histopathology 1991 19 403 410 10.1111/j.1365-2559.1991.tb00229.x 1757079

[B27] Cheang MC Chia SK Voduc D Gao D Leung S Snider J Watson M Davies S Bernard PS Parker JS Perou CM Ellis MJ Nielsen TO Ki67 index, HER2 status, and prognosis of patients with luminal B breast cancer J Natl Cancer I 2009 101 736 750 10.1093/jnci/djp082 PMC2684553 19436038

[B28] Xue C Wang X Peng R Shi Y Qin T Liu D Teng X Wang S Zhang L Yuan Z Distribution, clinicopathologic features and survival of breast cancer subtypes in Southern China Cancer Sci 2012 103 1679 1687 10.1111/j.1349-7006.2012.02339.x 22625227 PMC3466418

[B29] El Fatemi H Chahbouni S Jayi S Moumna K Melhouf MA Bannani A Mesbahi O Amarti A Luminal B tumors are the most frequent molecular subtype in breast cancer of North African women: an immunohistochemical profile study from Morocco Diagn Pathol 2012 7 170 10.1186/1746-1596-7-170 23216981 PMC3538531

[B30] Amadori D Serra P Bravaccini S Farolfi A Puccetti M Carretta E Medri L Nanni O Tumedei MM Kahima J Masalu N Differences in biological features of breast cancer between Caucasian (Italian) and African (Tanzanian) populations Breast Cancer Res Treatin press 10.1007/s10549-014-2903-0 24658893

[B31] Preat F Simon P Noel Preat JC Differences in breast carcinoma immunohistochemical subtypes between immigrant Arab and European women Diagn Pathol 2014 9 26 10.1186/1746-1596-9-26 24495621 PMC3915228

[B32] Sorlie T Perou CM Tibshirani R Aas T Geisler S Johnsen H Hastie T Eisen MB Van de Rijn M Jeffrey SS Thorsen T Quist H Matese JC Brown PO Botstein D EysteinLønning P and Børresen-Daleb Gene expression patterns of breast carcinomas distinguish tumor subclasses with clinical implications Proc Natl Acad Sci U S A 2001 98 10869 10874 10.1073/pnas.191367098 11553815 PMC58566

[B33] Kurebayashi J Moriya T Ishida T Hirakawa H Kurosumi M Akiyama F Kinoshita T Takei H Takahashi K Ikeda M Nakashima K The prevalence of intrinsic subtypes and prognosis in breast cancer patients of different races Breast 2007 16 Suppl 2 72 77 10.1016/j.breast.2007.07.017 17714947

[B34] Bauer KR Brown M Cress RD Parise CA Caggiano V Descriptive analysis of estrogen receptor (ER)-negative, progesterone receptor (PR)-negative, and HER2-negative invasive breast cancer, the so-called triple-negative phenotype: a population-based study from the California cancer Registry Cancer Am Cancer Soc 2007 109 1721 1728 10.1002/cncr.22618 17387718

[B35] Haffty BG Yang Q Reiss M Kearney T Higgins SA Weidhaas J Harris L Hait W Toppmeyer D Locoregional relapse and distant metastasis in conservatively managed triple negative early-stage breast cancer J Clin Oncol 2006 24 5652 5657 10.1200/JCO.2006.06.5664 17116942

[B36] Kim MJ Ro JY Ahn SH Kim HH Kim SB Gong G Clinicopathologic significance of the basal-like subtype of breast cancer: a comparison with hormone receptor and Her2/neu-overexpressing phenotypes Hum Pathol 2006 37 1217 1226 10.1016/j.humpath.2006.04.015 16938528

[B37] Su Y Zheng Y Zheng W Gu K Chen Z Li G Cai Q Lu W Shu XO Distinct distribution and prognostic significance of molecular subtypes of breast cancer in Chinese women: a population-based cohort study BMC Cancer 2011 11 292 10.1186/1471-2407-11-292 21749714 PMC3157458

[B38] Ly M Antoine M Dembélé AK Levy P Rodenas A Touré BA Badiaga Y Dembélé BK Bagayogo DC Diallo YL Koné AA Callard P Bernaudin JF Diallo DA High incidence of triple-negative tumors in sub-saharan Africa: a prospective study of breast cancer characteristics and risk factors in Malian women seen in a Bamako university hospital Oncology 2012 83 5 257 263 10.1159/000341541 22964749

[B39] Agboola AJ Musa AA Wanangwa N Abdel-Fatah T Nolan CC Ayoade BA Oyebadejo TY Banjo AA Deji-Agboola AM Rakha EA Green AR Ellis IO Molecular characteristics and prognostic features of breast cancer in Nigerian compared with UK women Breast Cancer Res Treat 2012 135 555 569 10.1007/s10549-012-2173-7 22842985

[B40] Salhia B Tapia C Ishak EA Gaber S Berghuis B Hussain KH DuQuette RA Resau J Carpten J Molecular subtype analysis determines the association of advanced breast cancer in Egypt with favorable biology BMC Women’s Health 2011 11 44 21961708 10.1186/1472-6874-11-44 PMC3204283

[B41] Steward L Conant L Gao F Margenthaler JA Predictive factors and patterns of recurrence in patients with triple negative breast cancer Ann Surg Oncolin press 10.1245/s10434-014-3546-4 24558065

[B42] Yuan N Meng M Liu C Feng L Hou L Ning Q Xin G Pei L Gu S Li X Zhao X Clinical characteristics and prognostic analysis of triple-negative breast cancer patients Mol Clin Oncol 2014 2 245 251 24649341 10.3892/mco.2013.230 PMC3917786

[B43] Lin NU Vanderplas A Hughes ME Theriault RL Edge SB Wong YN Blayney DW Niland JC Winder EP Weeks JC Clinicopathologic features, patterns of recurrence, and survival among women with triple-negative breast cancer in the National Comprehensive Cancer Network Cancer 2012 118 5463 5472 10.1002/cncr.27581 22544643 PMC3611659

[B44] Sugianto J Sarode V Peng Y Ki-67 expression is increased in p16-expressing triple-negative breast carcinoma and correlates with p16 only in p53-negative tumors Hum Pathol 2014 45 4 802 809 10.1016/j.humpath.2013.11.013 24560018

[B45] Taucher S Rudas M Mader RM Gnant M Dusky P Bachleitner T Roka S Fitzal F Kandioler D Sporn E Friedl J Mittlböck M Jakesz R Do we need HER-2/neu testing for all patients with primary breast carcinoma? Cancer 2003 98 2547 2553 10.1002/cncr.11828 14669272

[B46] Cusumano PG Generali D Ciruelos E Manso L Ghanem I Lifrange E Jerusalem G Klaase J De Snoo F Stork-Sloots L Dekker-Vroling L Holzik ML European inter-institutional impact study of MammaPrint Breastin press 10.1016/j.breast.2014.02.011 24685596

[B47] Drukker CA Van den Hout HC Sonke GS Brain E Bonnefoi H Cardoso F Goldhirsch A Harbeck N Honkoop AH Koornstra RHT Van Laarhoven HWM Portielje JEA Schneeweiss A Smorenburg CH Stouthard J Linn SC Schmidt MK Risk estimations and treatment decisions in early stage breast cancer: agreement among oncologists and the impact of the 70-gene signature Eur J Cancer 2014 50 1045 1054 10.1016/j.ejca.2014.01.016 24529927

